# Haematological parameters and their correlation with the degree of malaria parasitaemia among outpatients attending a polyclinic

**DOI:** 10.1186/s12936-023-04710-3

**Published:** 2023-09-25

**Authors:** Samuel Antwi-Baffour, Benjamin Tetteh Mensah, George Johnson, Dorinda Naa Okailey Armah, Samira Ali-Mustapha, Lawrence Annison

**Affiliations:** 1 Department of Medical Laboratory Sciences, School of Biomedical and Allied Health Sciences, College of Health Sciences, University of Ghana, Korle-Bu, P. O. Box KB 143, Accra, Ghana; 2 Department of Maternal and Child Health, School of Nursing, University of Ghana, Legon, Ghana; 3 Department of Medical Laboratory Technology, School of Medical Sciences, Accra Technical University, Accra, Ghana

**Keywords:** Malaria, Parasitaemia, Haematological, *Plasmodium falciparum*, Thrombocytopenia, Leukocytosis, Leukopenia and lymphocytopenia

## Abstract

**Background:**

Malaria is a parasitic disease caused by various species of the blood parasite *Plasmodium;* of all the parasitic diseases, malaria has the highest prevalence and mortality with an estimated 247 million cases and 619,000 deaths recorded worldwide as of 2021. Malaria causes febrile illness with several changes in blood cell parameters. Some of these changes include leucopenia, thrombocytopenia, and anaemia. If these changes could be correlated with the degree of parasitaemia, it can serve as a guide to physicians when treating malaria. This study was therefore aimed at correlating haematological parameters with levels of parasitaemia during malaria infection.

**Methods:**

The study was a cross-sectional study involving 89 malaria positive patients. About 5 ml of blood was collected from each participant who gave his or her informed consent to partake in the study. A full blood count was performed on their samples to determine their haematological parameters using a haematology auto-analyzer. A parasite count was also performed via microscopy to determine the degree of parasitaemia. The data obtained from the study was entered into a database and statistically analysed using Statistical Package for Social Sciences (SPSS) version 23 and Microsoft Excel 2016.

**Results:**

The study comprised of 89 participants out of which 35 were males and 54 were females with the mean age of 26.15 years. Secondary education participants were the highest with quaternary education the lowest. The highest parasite count recorded was 398,174 parasites/µl of blood, lowest count was 101 with the average being 32,942.32584. There was also a significant positive Pearson’s correlation between total WBC and parasitaemia and with the WBC differentials, neutrophils, lymphocytes and monocytes had positive correlations while eosinophils and basophils had negative correlations. Furthermore, platelets, total RBC’s, haemoglobin, MCH, MCHC and Hct all showed negative correlations. Linear regression also showed a linear relationship between parasite density and the various haematological parameters.

**Conclusion:**

The linear relationship (correlation) between WBC and MCH were the only significant ones at 95% and 99% confidence interval, respectively based on a two-tail t-test. Also, based on the regression analysis, the changes caused by WBC and PLT were the only significant changes at 95% confidence level in a two-tailed t-test.

## Background

Malaria is a life-threatening disease that is caused by parasites transmitted to people through the bites of infected female *Anopheles* mosquitoes [1]. The infection may result in a wide variety of symptoms, ranging from absent or very mild symptoms to severe disease and even death [1, 2]. There are five parasite species that cause malaria in humans, and two of these—*Plasmodium falciparum* and *Plasmodium vivax*—pose the greatest threat [1]. Malaria infections occur in five WHO regions and globally an estimated 3.4 billion people in 91 countries and territories are at risk of being infected with malaria and developing the disease with additional 1.1 billion at high risk [3]. In 2021, there were an estimated 247 million cases in malaria and 619,000 deaths worldwide [1]. Malaria is mainly endemic in tropical regions and developing countries due to the poor sanitation conditions in these developing countries [4]. In Ghana, 3.5 million cases of malaria are reported annually [5].

All of the clinical symptoms we see in malaria infection are caused by the asexual erythrocytic or blood stage parasites [6]. As the parasite develops in the erythrocyte, numerous waste substances that may be known or unknown, such as haemozoin pigment and other toxic factors accumulate in the infected erythrocyte [7]. These are released into the bloodstream when the infected cells lyses and releases merozoites that invade other red blood cells [7]. The haemozoin and the other toxic factors, such as glucose phosphate isomerase (GPI) stimulate macrophages as well as other cells to produce signaling molecules and other soluble factors which together act to produce fever and rigors and may even influence other severe pathophysiology associated with malaria [8, 9]. Furthermore, *P. falciparum-*infected erythrocytes, particularly those with mature trophozoites, adhere to the vascular endothelium of venular blood vessel walls and do not freely circulate in the blood [10]. When this sequestration of infected erythrocytes occurs in the vessels of the brain, it leads to cerebral malaria, which is associated with high mortality particularly in children [11].

Malaria causes acute febrile illness and is often difficult to differentiate from other febrile illnesses such as leptospirosis and arboviral infection hence it might not be detected early in patients resulting in several complications [12]. Symptoms associated with malaria include diarrhoea, vomiting, myalgia and abdominal pain. However, life-threatening complications such as kidney and liver failure, anaemia, low blood sugar, cerebral malaria and pulmonary oedema may also occur [13]. It must also be mentioned that, the severity of the disease is related to the level of parasitaemia, with high levels of parasitaemia resulting in severe malaria. Consequently, a sample of blood with 5–10,000 parasites/µl could be considered as having a low parasitaemia resulting in mild malaria, and one with 10,000–100,000 parasites/µl could be considered as having intermediate parasitaemia resulting in moderate malaria, whilst parasitaemia above 100,000 parasites/µl is said to be hyperparasitaemia and may result in severe malaria that may further result in death [14]. Generally, the severity of disease and risk of mortality in malaria would be expected to be dependent on the degree of parasitaemia [15].

Furthermore, when one is infected with malaria, various haematological alterations such as anaemia, thrombocytopenia, leukocytosis or leukopenia do occur [16, 17]. Lymphocytopenia has also frequently been described in patients with malaria, but studies on its association with disease severity have yielded conflicting results [18]. It has been postulated that the variation in parasitaemia levels may cause similar variation in haematological parameters [15]. That is to say, the changes in the haematological parameters may vary in relation with the levels of parasitaemia. Parasitaemia is highest in *P. falciparum* infections since the parasite invades red blood cells of all ages hence causing the most severe infections [19]. Due to this and since Ghana has more *P. falciparum* infections, it would be vital if a correlation between haematological parameters and parasitaemia could be established as it will enable physicians to determine how to proceed with early treatment and management of malaria.

## Methods

### Aim, design and setting of study

The aim of this study was to correlate changes in blood cell parameters with levels of parasitaemia in malaria infection. This study was a cross sectional study conducted from January 2018 to December 2018 at the Mamprobi polyclinic in Accra, Ghana.

### Participants

The study comprised of eighty-nine (89) male and female patients attending the polyclinic and diagnosed with malaria infection and who gave their written informed consent.

### Data/sample collection and laboratory analysis

Three to five millilitres (3–5 ml) of blood samples were collected from a prominent vein of each participant by venipuncture into lavender-topped tubes containing Ethylene-Diamine-Tetra-Acetic acid (EDTA) and thoroughly mixed to prevent them from clotting. The patients' unique identification numbers on consent forms were written on each tube to ensure confidentiality. A haematology analyzer was then used to perform a full blood count on the patients' blood samples for their haematological parameters. Thick and thin blood films were also prepared using the same patients' blood samples. The thick films were stained with Giemsa and the thin films with Leishman stain. The thick films were then examined under × 100 magnification for malaria parasites identification and the thin films for parasite specie identification. To determine the parasite density, two hundred (200) leukocytes were first counted whilst observing the malaria parasites present. If less than 10 parasites were observed after counting 200 leukocytes the count was continued till 500 leukocytes were counted. The parasite density was then calculated using the formula below.

$$\frac{{{\text{Number}}\;{\text{of parasites}}}}{{{\text{Number}}\;{\text{of leukocytes}}}} \times 8000.$$

This gave the number of parasites/µl of blood.

### Data analysis

Data obtained were analyzed using Microsoft Excel 2016 and Statistical Package for Social Sciences (SPSS) software Version 23 (SPSS Inc., Chicago, USA). Descriptive statistics, Correlation analysis and linear regression analysis were carried out on the data.

## Results

### Demographics of participants

There were eighty-nine (89) participants in the study out of which 35 were males and 54 were females representing 39% and 61%, respectively. The oldest participant was 78 years while the youngest was 9 months. The mean age recorded was 26.15 years. With regards to educational background, 13 participants had none, 15 had primary, 43 had secondary, 16 had tertiary and 2 had quaternary education (Table 1).

**Table 1:** Descriptive statistics of demographics of participants

Age (years)	
Mean	26.1544944
Standard error	2.14658309
Median	22
Mode	18
Standard deviation	20.2508244
Sample variance	410.095889
Kurtosis	− 0.674499
Skewness	0.59413663
Range	77.25
Minimum	0.75
Maximum	78
Sum	2327.75
Count	89
Gender	
Male	35 (39%)
Female	54 (61%)
Education	
None	13 (14.6%)
Primary	15 (16.9%)
Secondary	43 (48.3%)
Tertiary	16 (18.0%)
Quaternary	2 (2.2%)

### Parasite indentification and density measurement from participants

Only *P. falciparum* species were identified. In terms of the parasite count, the highest parasite count was 398,174 parasites/µl whilst the lowest was 101 parasites/µl and the average parasite count recorded was 32,942.32584 parasites/µl. Also, in terms of gender distribution, males had the highest average parasite count whilst females had the lowest as seen in Table 2.

**Table 2:** Parasite density of participants

Parasite density	Parasites/µl
Mean	32,942.32584
Standard error	7176.86209
Median	6014
Mode	504
Standard Deviation	67,706.38154
Sample variance	4,584,154,102
Kurtosis	16.92672812
Skewness	3.902153243
Range	398,073
Minimum	101
Maximum	398,174
Sum	2,931,867
Count	89
Gender	
Male (mean)	42,000.25183
Female (mean)	28,000.16525

**Fig. 1:**
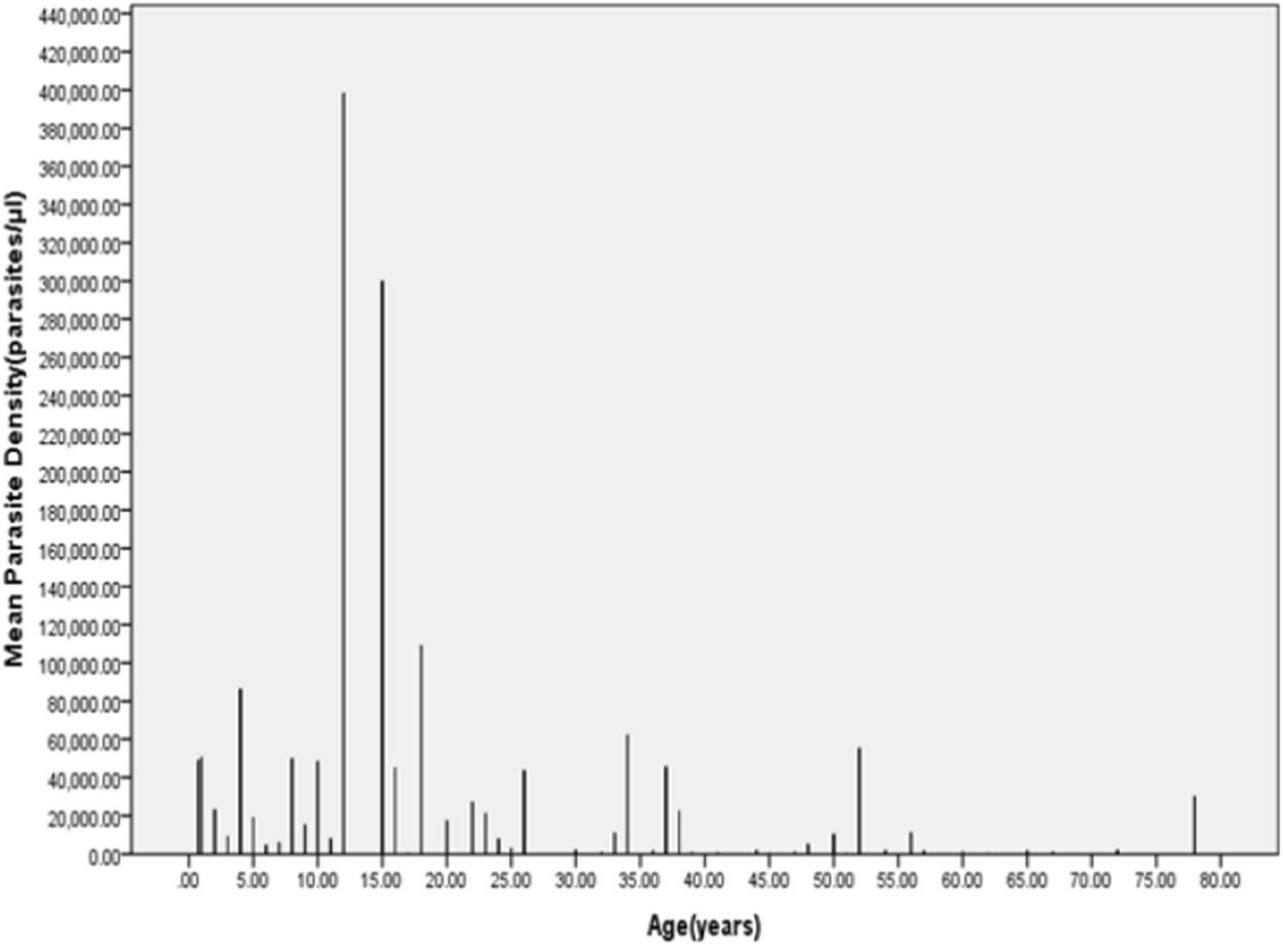
Bar graph of parasite density against age

The mean parasite density were also compared with age of participants and the outcome is shown in Fig. 1.

### Correlation between parasitaemia and various haematological parameters

Pearson’s correlation was run between parasitaemia and various haematological parameters and significant positive correlation was found between total WBC and parasitaemia. On the part of the WBC differentials, neutrophils, lymphocytes and monocytes had positive correlations while eosinophils and basophils had negative correlations. Specifically, it was seen that for 1 unit (parasites/µl) rise in parasite density there was 0.300 units (WBC × 109/l) rise in WBC and of the five white cells, the neutrophils were the most responsible for this rise by 0.297 units (Neu × 109/l) followed by monocytes and lymphocytes with a rise of 0.053 and 0.014 units respectively. Eosinophils and basophils fell by 0.021 and 0.025 units, respectively anytime parasitaemia rose by 1 unit (Table 3).

**Table 3:** Correlation between parasitaemia, WBC and its differentials

	Parasite density	WBC	Neutrophils	Lymphocytes	Monocytes	Eosinophils	Basophils
Parasite density							
Pearson Correlation	1	0.300^**^	0.297^**^	0.014	0.053	− 0.021	− 0.025
Sig. (2-tailed)		0.004	0.005	0.893	0.623	0.843	0.817
N	89	89	89	89	89	89	89
WBC							
Pearson correlation	0.300^**^	1	0.771^**^	0.403^**^	0.310^**^	0.002	0.227^*^
Sig. (2-tailed)	0.004		0.000	0.000	0.003	0.985	0.032
N	89	89	89	89	89	89	89
Neutrophils							
Pearson Correlation	0.297^**^	0.771^**^	1	− 0.218^*^	0.058	− 0.108	− 0.064
Sig. (2-tailed)	0.005	0.000		0.041	0.590	0.313	0.552
N	89	89	89	89	89	89	89
Lymphocytes							
Pearson Correlation	0.014	0.403^**^	− 0.218^*^	1	0.187	0.097	0.249^*^
Sig. (2-tailed)	0.893	0.000	0.041		0.079	0.367	0.019
N	89	89	89	89	89	89	89
Monocytes							
Pearson Correlation	0.053	0.310^**^	0.058	0.187	1	0.042	0.371^**^
Sig. (2-tailed)	0.623	0.003	0.590	0.079		0.693	0.000
N	89	89	89	89	89	89	89
Eosinophils							
Pearson Correlation	− 0.021	0.002	− 0.108	0.097	0.042	1	0.094
Sig. (2-tailed)	0.843	0.985	0.313	0.367	0.693		0.380
N	89	89	89	89	89	89	89
Basophils							
Pearson Correlation	− 0.025	0.227^*^	− 0.064	0.249^*^	0.371^**^	0.094	1
Sig. (2-tailed)	0.817	0.032	0.552	0.019	0.000	0.380	
N	89	89	89	89	89	89	89

**Correlation is significant at the 0.01 level (2-tailed)

*Correlation is significant at the 0.05 level (2-tailed)

Also, Haemoglobin and RBC both had negative correlations and reduced by 0.205 and 0.062 units respectively per unit increase in parasitaemia. Haematocrit also had a negative correlation thus dropping by 0.142 units for every unit increase in parasite density. MCH and MCHC also had negative correlations of − 0.240 and − 0.040, respectively. There was also a 0.142 reduction in platelet levels per every unit increase in parasite density (Table 4).

**Table 4:** Correlation between parasitaemia, platelets, Hb, RBC and RBC indices

Correlations								
		Parasite density	Haemoglobin	HCT	MCH	MCHC	Platelet	RBC
Parasite Density	Pearson Correlation	1	− 0.205	− 0.142	− 0.240^*^	− 0.040	− 0.142	− 0.062
	Sig. (2-tailed)		0.054	0.185	0.024	0.708	0.184	0.564
	N	89	89	89	89	89	89	89
Haemoglobin	Pearson Correlation	− 0.205	1	0.880^**^	0.297^**^	− 0.104	− 0.225^*^	0.764^**^
	Sig. (2-tailed)	0.054		0.000	0.005	0.330	0.034	0.000
	N	89	89	89	89	89	89	89
HCT	Pearson Correlation	− 0.142	0.880^**^	1	0.087	− 0.486^**^	− 0.163	0.799^**^
	Sig. (2-tailed)	0.185	0.000		0.418	0.000	0.126	0.000
	N	89	89	89	89	89	89	89
MCH	Pearson Correlation	− 0.240^*^	0.297^**^	0.087	1	0.471^**^	− 0.089	− 0.346^**^
	Sig. (2-tailed)	0.024	0.005	0.418		0.000	0.409	0.001
	N	89	89	89	89	89	89	89
MCHC	Pearson Correlation	− 0.040	− 0.104	− 0.486^**^	0.471^**^	1	− 0.001	− 0.407^**^
	Sig. (2-tailed)	0.708	0.330	0.000	0.000		0.994	0.000
	N	89	89	89	89	89	89	89
Platelet	Pearson Correlation	− 0.142	− 0.225^*^	− 0.163	− 0.089	− 0.001	1	− 0.136
	Sig. (2-tailed)	0.184	0.034	0.126	0.409	0.994		0.204
	N	89	89	89	89	89	89	89
RBC	Pearson Correlation	− 0.062	0.764^**^	0.799^**^	− 0.346^**^	− 0.407^**^	− 0.136	1
	Sig. (2-tailed)	0.564	0.000	0.000	0.001	0.000	0.204	
	N	89	89	89	89	89	89	89

*Correlation is significant at the 0.05 level (2-tailed)

**Correlation is significant at the 0.01 level (2-tailed)

### Linear regression between parasitaemia and haematological parameters

**Fig. 2:**
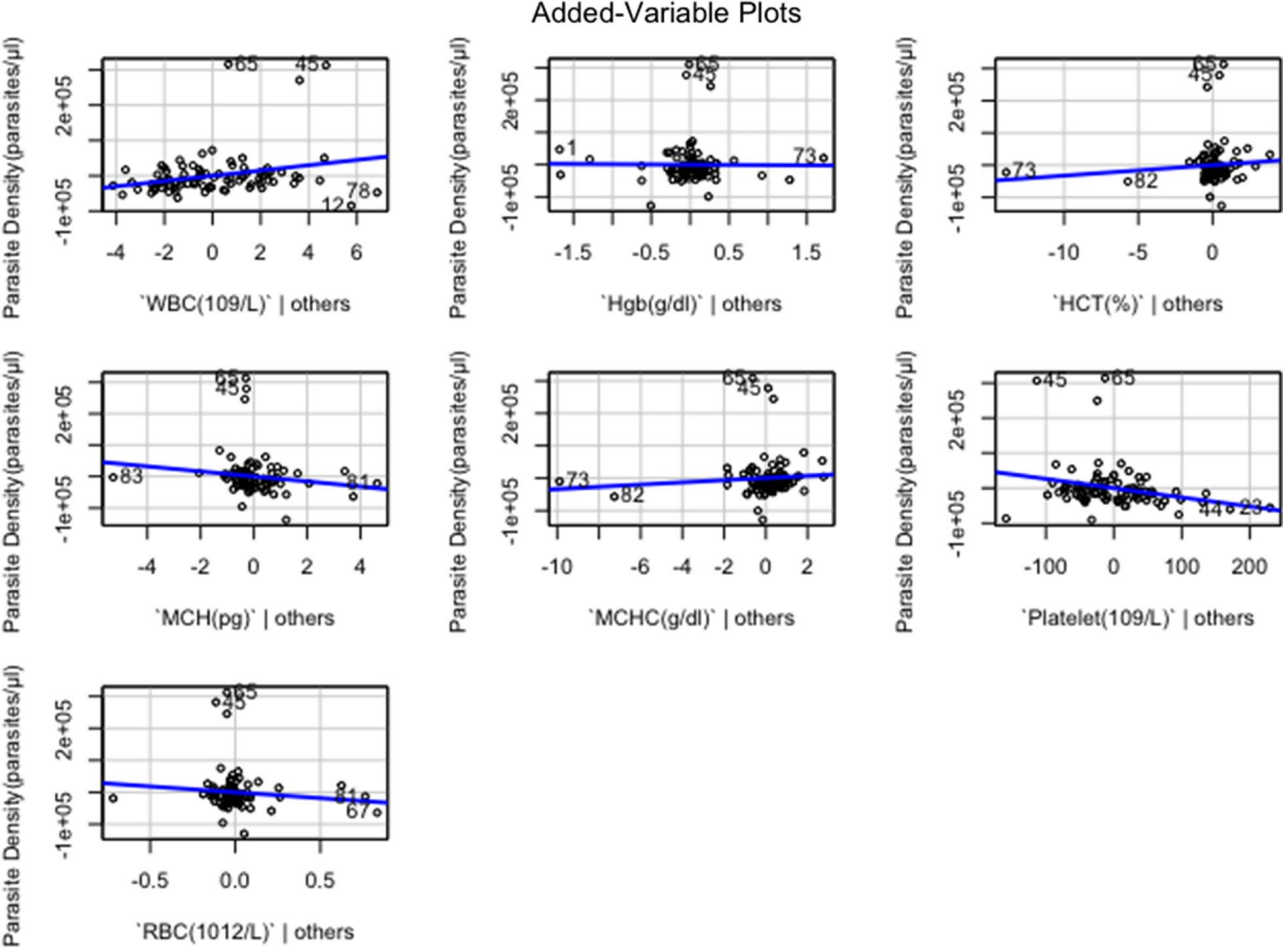
Fitted line plots (linear slopes) for regression coefficient interpretation

A regression analysis was performed to ascertain the relationship between selected haematological parameters (WBC, Hgb, HCT, MCH, MCHC, Platelet and RBC) and parasitaemia. The model had parasitaemia as the outcome variable and the various haematological parameters as predictor variables. A multiple R of 0.44 (correlation between the outcome and predictor variables) was obtained. This suggests a moderate degree of association. The R-squared of 0.195 implies that approximately 19.5% of variations in parasitaemia (outcome variable) can be explained by the selected Haematological parameters (predictor variables). However, the model’s adjusted R-squared value which explains variation in outcome variables caused by predictor variables while adjusting for the number of predictor variables was relatively low (0.125). This indicates that other unmeasured factors might also be influencing the outcome variable (Fig. 2).

An ANOVA was also performed to further understand the model’s fit and significance. The results of the test inferred that the regression model as a whole was statistically significant as evidenced by the F-statistic of 2.797 (p-value = 0.1117). This indicated that the predictor variables had a significant influence on predicting the outcome variable. The significant F-statistic and corresponding p-value supported the model's overall fit. Notwithstanding, only WBC and platelet counts showed statistically significant relationships with the outcome variable. The positive coefficient for WBC indicates that an elevation in WBC is associated with an increase in the outcome variable. In contrast, the negative Platelet coefficient indicates that a decrease in Platelet count is associated with a decline in the outcome variable. All the other predictor variables (Hgb, HCT, MCH, MCHC, and RBC) did not show statistically significant associations with parasitaemia.

## Discussion

In this study eighty-nine malaria patients were recruited, and the majority of participants (61%) were females, and the rest (39%) were males. The average parasite count of the participants was 32,942.32584 parasites/µl with the lowest being 101 parasites/µl and the highest of 398,174 parasites/µl. The average parasite count of the males (42,000 parasites/µl) was 50% higher than that of their female counterparts (28,000 parasites/µl) although, the female participants outnumbered the males in the study. The age range of the participants was between 9 months and 78 years with the mean age being 26.15 years. The age with the highest parasitaemia was 12 while that with the lowest was 62. Also, most of the WBC counts were either within the normal range or lower than the reference but there were few cases of leukocytosis. The haemoglobin, platelet and other RBC indices also showed variations as parasitaemia increased. This clearly demonstrates that there is a relationship between the degree of parasitaemia and various haematological parameters.

After the analysis, correlations were established between parasitaemia and various haematological parameters. There was a positive correlation between parasitaemia and WBC count.

Eosinophils and basophils are the least numerous WBCs in circulation and often associated with allergic reactions thus this might be a reason for their negative correlation. Also, haemoglobin and RBC both had negative correlations as parasitaemia increased. This could be due to the fact that as the *Plasmodium* parasite resides in RBCs, their activities results in the lyses of the cells thus reducing their numbers leading to haemolytic anaemia in most malaria infection cases [20].

MCH and MCHC, which are dependent on RBC, Hb and Hct values also showed negative correlations. There was also a reduction in platelet levels per every unit increase in parasite density, which could be due to the immune and non-immune destruction of platelets during malaria infection. The exact course of platelet destruction is not known but some of the assumed mechanisms may include coagulation disturbances, sequestration in spleen, antibody mediated platelet destruction, oxidative stress, and the role of platelets as cofactors in triggering severe malaria [21].

Again, the regression relationship between RBC, Hb and MCH with parasite density were negative while that of MCHC and Hct were positive. However, these relationships were insignificant based on a two-tail t-test performed at 95% confidence level. The findings of this study are similar to that of Wang and Xing who posited that interactions between *Plasmodium* infested RBCs and normal RBCs can result in fluctuations in Hct [22]. Similarly, Maina et al*.* found that platelets, lymphocytes, eosinophils, red blood cell count and haemoglobin were significantly lower in malaria-infected children [23]. Again, Akhtar et al*.* also carried out a study where they showed that 70% of the patients with malaria had thrombocytopenia, 94% anaemia, 12% lymphopenia and 17% monocytosis [24]. The current study is also in line with the work of Al-Salahy et al*.* who found that neutrophils levels were significantly higher in cases of falciparum malaria in comparison to healthy normal subjects [25].

It is worthy to mention that red blood cells infected with *P. falciparum* malaria parasite are known to adhere to endothelial cells lining blood vessels [26]. This phenomenon, also known as sequestration is associated with a number of features seen in severe malaria infection such as cerebral malaria and pregnancy-associated malaria [27]. This adherence of infected red blood cells, occurs in small capillaries and post-capillary venules of specific organs such as the brain and lungs [28]. Indeed the sequestration of malaria parasite infected RBCs has been correlated with mechanical obstruction of blood flow in small blood vessels and vascular endothelial cell activation, which may lead to serious pathology [29]. In terms of parasitaemia, the sequestration may affect the level negatively causing less number of parasites to be seen in peripheral blood as compare with the actual number in the individual. Sequestration may, therefore, results in low levels of parasitaemia. A limitation worthy of mention is that, not many malaria cases were seen at the clinic during the period of the study and some of the patients were also not willing to take part in the study. However, the number of participant needed for the study was obtained.

## Conclusion

The outcome of the study indicates that there exists a correlation between the various haematological parameters and degree of parasitaemia. However, upon performing two-tail t-test to determine the significance of the correlation at 95% and 99% confidence level, it was concluded that only WBC and MCH had a significant linear relationship (correlation) with the degree of parasitaemia at 95% and 99% confidence level, respectively. Regression analysis also showed that the variation in parasitaemia (dependent variable) resulted in changes in the various haematological parameters (independent variables). Howbeit, only the changes caused by WBC and PLT were significant at 95% confidence level in a two-tailed t-test.

## Data Availability

The datasets used and/or analyzed during the current study are available from the corresponding author on reasonable request.

## Abbreviations

WBC: White blood cell
PLT: Platelet
MCH: Mean cell haemoglobin
RBC: Red blood cell
MCHC: Mean cell haemoglobin concentration
Hct: Haematocrit
Hb: Haemoglobin
WHO: World Health Organization
GPI: Glucose phosphate isomerase
